# Ring Structures of Metal Atom Doped C_9_, C_11_, and C_13_ Clusters from Ab Initio Calculations—The Finding of a Gd@C_13_ Magnetic Superatom Ring

**DOI:** 10.1021/acsomega.4c04141

**Published:** 2024-07-26

**Authors:** Vijay Kumar

**Affiliations:** † Center for Informatics, School of Natural Sciences, Shiv Nadar Institution of Eminence, NH-91, Tehsil Dadri, Gautam Buddha Nagar, Greater Noida, Uttar Pradesh 201314, India; ‡ Dr. Vijay Kumar Foundation, 1969, Sector 4, Gurgaon, Haryana 122001, India

## Abstract

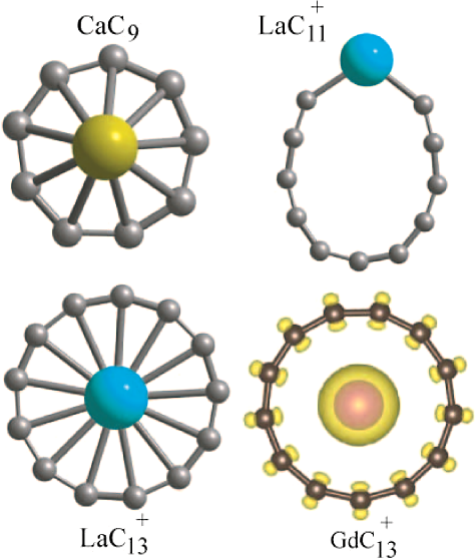

Pure C_9_ clusters have a linear chain structure. However, here, we report using ab initio calculations the transformation of a chain into a cyclic ring structure with the capping of Ca, Sr, and Ba atoms. Further calculations on neutral and charged clusters doped with Sc, Y, and La atoms show stabilization of a cation C_9_ isoelectronic cyclic ring capped with the metal (M) atom, but anion clusters doped with these trivalent atoms form a C_10_ like MC_9_ ring, which deforms to a necklace structure. Both the ring structures correspond to electronic shell closing with 20 delocalized valence electrons in a disk jellium model and have a large highest occupied molecular orbital–lowest unoccupied molecular orbital (HOMO–LUMO) gap. Calculations of IR and Raman spectra show no imaginary frequency, suggesting that the structures are stable. Addition of two C atoms to the ring structure of LaC_9_ leads to a capped ring structure of the cation LaC_11_ and an open ring structure of the LaC_11_ anion. Further addition of two C atoms leads to a La@C_13_ cation as well as Ca@C_13_ and Sr@C_13_ neutral wheel-shaped rings with endohedral doping of the M atom. These novel ring structures have a large HOMO–LUMO gap of more than 4 eV and are magic with electronic shell closing corresponding to 28 delocalized valence electrons in a disk jellium model. There is π aromaticity in this ring satisfying 4*n*+2 (*n* = 3) Hückel’s rule with 14 valence electrons. Interestingly, when the dopant is a Gd atom, there is a formation of a magnetic superatom ring Gd@C_13_^+^ with 7 μ_B_ magnetic moments due to seven 4*f* up-spin states of Gd being fully occupied. Bonding in these novel ring structures is discussed.

## 1 Introduction

Clusters and nanostructures of carbon have attracted great interest in the past few decades with path breaking discoveries of fullerenes,^1^ nanotubes,^2^ and graphene.^3^ Doping of metal (M) atoms in these nanostructures has also attracted much interest in manipulating their properties for different applications including catalysis, sensors, and functionalized materials. This has led to the discovery of endohedral fullerenes^4^ with an M atom or a group of atoms inside a fullerene cage. In these doped species, generally, no significant change occurs in the atomic structure of the host cluster or the nanostructure due to doping. However, in other related systems such as clusters of silicon and Ge, the doping of an M atom led to the finding of novel structures^5−8^ with very different atomic arrangements compared with those of the bulk or undoped clusters. Even the formation of nanotubes^9−11^ of silicon has been shown with the doping of M atoms. An interesting situation arises when the number of carbon atoms in a fullerene reduces to less than 30. With the shrinking of the cage, the bonding develops significant *sp*^3^ character due to the reduction in the number of hexagons, and in the smallest fullerene C_20_ with all pentagonal faces, the bonding becomes close to *sp*^3^ type. This leads to lower stability of the cage due to the dangling bonds. It has been found^7,12−14^ that the stability of such small carbon fullerenes with 20–28 atoms can be enhanced with the doping of an M atom as in the case of endohedrally doped silicon fullerenes and other polyhedral structures.^15^ In these cases, the M atom interacts strongly with the silicon and carbon fullerene cages. We ask the following question: what will happen to the structures if the number of carbon atoms is reduced further to less than 20 and if their structures can be manipulated with the doping of an M atom?

Carbon clusters with less than 20 atoms have a sudden change in the structure with rings becoming more favorable.^16,17^ Currently, these systems are attracting much interest with reports of the formation of C_10_, C_14_, C_16_, and C_18_ rings^18−20^ on a substrate. Clusters with less than 10 carbon atoms and particularly those with an odd number of atoms such as C_7_ and C_9_ have a chain structure.^21^ Doping of an atom in some small clusters of carbon such as CuC_10_, SiC_9_, and so on has been studied,^22,23^ but the structures of the doped clusters remain similar to those of the pristine ones. In a study^24^ on La doped carbon cation clusters, mobility experiments suggested the formation of M atom doped ring structures with varying number of carbon atoms, although no theoretical understanding or confirmation was achieved. It is of interest to explore the doping of M atoms in small carbon clusters – an area that is still not well studied – and to search if there could be new forms of carbon. Very recently, doping of an M atom (M = Be, Mg, and Al) in the C_9_ cluster has been shown^25^ to transform a chain structure of C_9_ to a ring structure. Here, we study doping of a C_9_ cluster with Ca, Sr, and Ba atoms and show that these doped clusters favor a ring structure of carbon with the M atom capping the ring in contrast to M = Be and Al for which the M atom is part of the ring. The choice of a divalent atom emerged from the fact that the LUMO and LUMO+1 states of a C_9_ ring have large energy gap. The LUMO can be occupied if a divalent electropositive atom is added, leading to the possibility of a highly stable doped C_9_ ring. Furthermore, we considered doping of Sc, Y, and La atoms as well as their anion and cation species. Our results show that the cations of Sc, Y, and La doped clusters are isoelectronic to those doped with a divalent M atom and have a cyclic ring structure with the M atom capping the ring, but the anion of all the three trivalent M doped clusters stabilizes in another ring structure in which the M atom is incorporated in a ring similar to a highly stable C_10_ ring. In fact, the anions of these doped clusters become electronically equivalent to C_10_ with the same number of valence electrons, but the optimized ring relaxes to a necklace shape. By further adding two C atoms, we formed a MC_11_ (M is a trivalent atom) ring, which is favored by the cation, but for the La doped anion, the ring becomes open. Therefore, we added two more C atoms and found a novel La@C_13_ cation cyclic ring with the La atom endohedrally doped inside the C_13_ ring. Similar results have been obtained for the isoelectronic Ca and Sr doped C_13_ neutral rings, while Ba sits slightly above the ring. There is a large HOMO–LUMO gap in these cases suggesting the chemical stability of these rings with a closed electronic shell. As rare earth atoms also behave as trivalent, we further considered M as a Gd atom. Interestingly, it forms a Gd@C_13_^+^ magnetic superatom ring with half-filled 4*f* levels and 7 μ_B_ magnetic moments.

## 2 Results and Discussion

**Figure 1:**
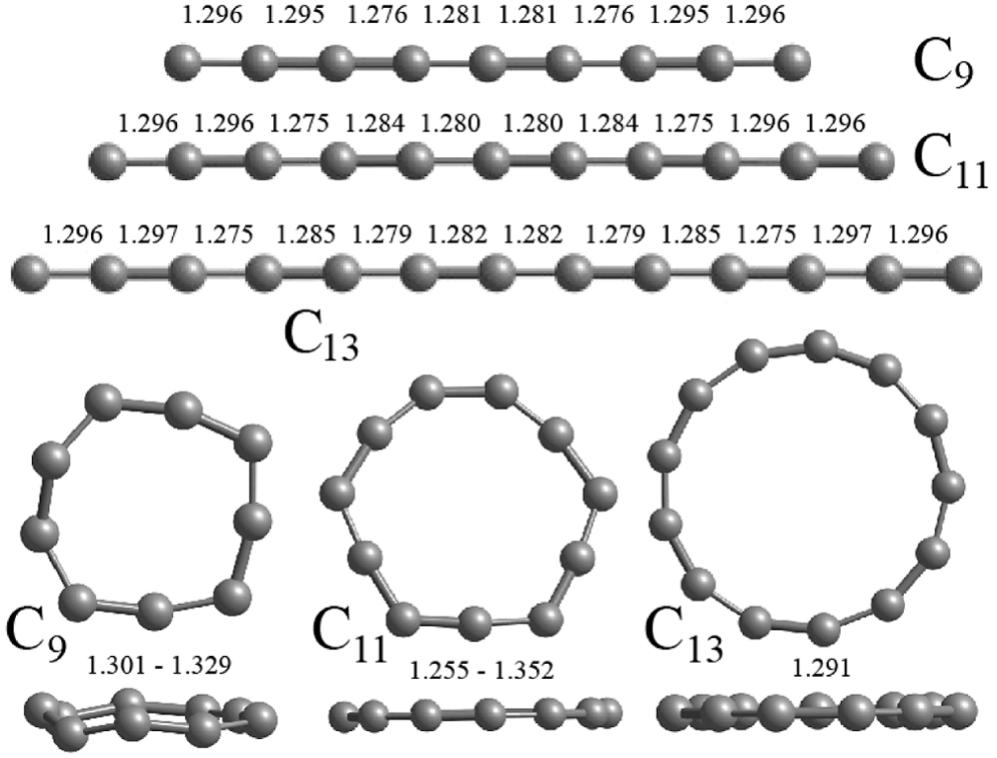
Optimized atomic structures of chain and ring isomers of C_9_, C_11_, and C_13_ clusters. For C_9_, the chain isomer lies 0.435 eV lower in energy than the ring isomer, while for C_11_ and C_13_, the ring isomer lies 0.935 and 1.124 eV lower in energy, respectively, than the chain isomer using PBE. For the ring isomers, we have also shown the side view. It is seen that the ring isomer of C_9_ is buckled, but for C_11_ and C_13_, all atoms lie in a plane. The C_13_ ring has 2 μ_B_ magnetic moments and all the bond lengths are equal (1.291 Å), while in other cases, there are small variations and the magnetic moment is zero. The bond lengths are given in Å.

The optimized chain and ring structures of the pure C_9_ cluster are shown in Figure 1. The chain structure lies^25^ 0.435 eV (0.250 eV) lower in energy compared with a ring structure using PBE (PBE0). There is a small variation in the C–C bond lengths (1.296 Å at the ends, 1.295, 1.276, and 1.281 Å in the middle of the chain using PBE as shown in Figure 1), and the magnetic moment is zero. The bond lengths become slightly contracted (1.283 Å at the ends, 1.286, 1.267, and 1.273 Å in the middle) when PBE0 is used. The HOMO–LUMO gap is 1.715 eV (2.978 eV) using PBE (PBE0). The ring structure is slightly buckled and there is a small variation in the nearest neighbor C–C bond lengths, which lie in the range of 1.301–1.329 Å (1.286–1.322 Å) using PBE (PBE0). These results show a slight increase in the bond lengths in the ring isomer compared with the chain. The ring isomer also has 0 μ_B_ magnetic moment, and the HOMO–LUMO gap is smaller [0.648 eV (2.378 eV) using PBE (PBE0)] compared with that of the chain. It is also much smaller than 3.750 eV (5.70 eV) obtained for a C_10_ 5-fold symmetric ring within the PBE (PBE0). The large value of the HOMO–LUMO gap for C_10_ makes it a particularly stable ring. All of the nearest neighbor bond lengths for C_10_ are equal to 1.295 (1.288 Å) using PBE (PBE0) and are slightly smaller than the values for C_9_. We also calculated the C_9_ anion using the Gaussian code, and the chain structure is found to be 1.016 eV lower in energy than a ring isomer. This result agrees with experiments^16^ where a chain of C_9_ anion has been reported. For C_11_ and C_13_, the converged chain and ring structures are also shown in Figure 1 and the ring structures are 0.935 and 1.124 eV lower in energy, respectively, than the chain structure using PBE. The C_13_ ring has 2 μ_B_ magnetic moments while the C_11_ ring is nonmagnetic. The bond lengths in C_11_ ring lie in the range of 1.255–1.352 Å using PBE (see Figure 1) while the HOMO–LUMO gap is 0.57 eV. However, using PBE0, the HOMO–LUMO gap is 2.114 eV. The atomic structure of the C_11_ ring can be derived from a C_12_ hexagon by removing an atom. For C_13_, the ring is symmetric. All of the bond lengths are equal (1.291 Å using PBE) and are slightly shorter than those of C_10_. The HOMO–LUMO gap is 0.246 eV within PBE, but it is 2.055 eV using PBE0. The C_13_ chain isomer, however, shows variation in the bond lengths (Figure 1). We find an oscillatory behavior of the bond lengths in chain isomers. We expect only a small difference in the bond lengths calculated by using PBE and PBE0, but the HOMO–LUMO gap is significantly underestimated in PBE.

**Figure 2:**
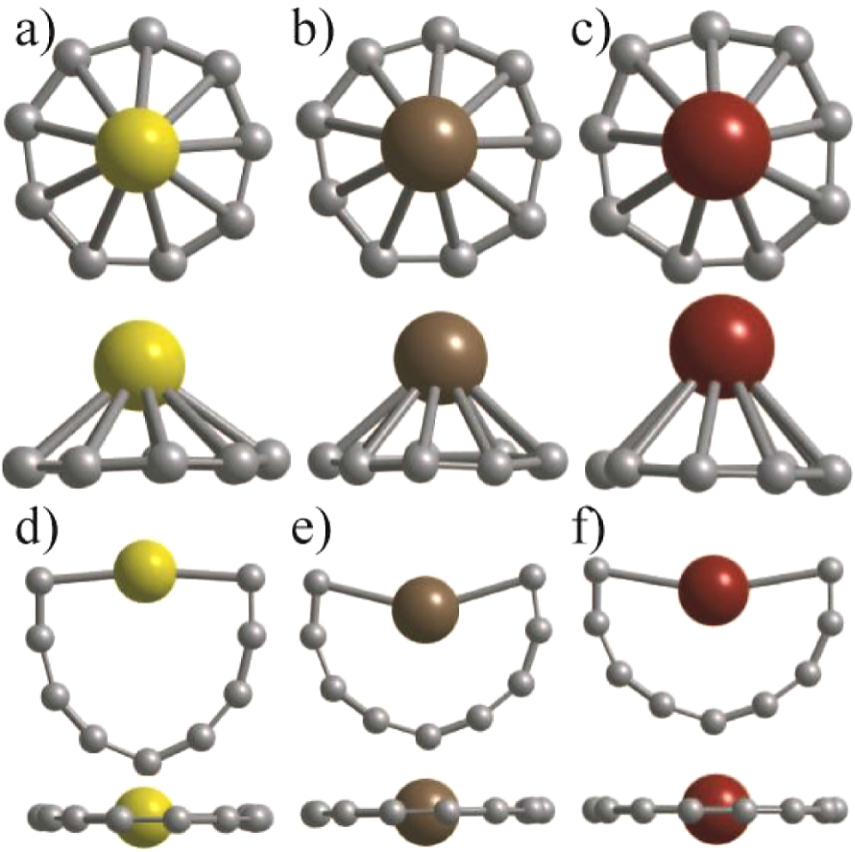
Capped ring and ring (necklace) structures of (a,d) CaC_9_, (b,e) SrC_9_, and (c,f) BaC_9_, respectively. The capped ring structures have the lowest energy. Both the top and side views are shown. For Mn doping, the structures are similar to those in Figure 2a,d with the ring isomer lying lower in energy than the capped ring. The large (small) balls show M (C) atoms in each case.

We further added an M atom at one of the ends of the C_9_ chain, capped the C_9_ ring, and inserted it in the ring to make a MC_9_ ring. The optimized structures for the M = Ca, Sr, and Ba atom doped systems are shown in Figure 2. It is found that for these M atoms, the capped ring (Figure 2a–c) structure has the lowest energy. This is different from the case^25^ of M = Be and AlC_9_ cation cluster. There is a small variation in C–C bond lengths in the C_9_ ring, which vary in the range of 1.27–1.34 Å for M = Ca, while the C–Ca bond lengths vary in the range of 2.41–2.48 Å using PBE. There is a kind of pentagonal distortion in the ring with a mirror symmetry in a plane perpendicular to the ring. Similar results have been obtained for Sr (Ba) with the C–C bond lengths lying in the range of 1.27–1.35 Å (1.28–1.35 Å) and Sr–C (Ba–C) bond lengths lying in the range of 2.57–2.65 Å (2.74–2.83 Å). It is noted that the change in C–C bond lengths is quite small, but M–C bond lengths increase significantly in going from M = Ca to Sr and then to Ba due to the increasing size of the M atom. As there is a large gap between LUMO and LUMO+1 for the ring isomer of C_9_, the addition of a divalent electropositive M atom fills the LUMO due to the charge transfer (see below) and leads to a large HOMO–LUMO gap of 4.046, 3.821, and 3.908 eV for M = Ca, Sr, and Ba, respectively, in the capped ring isomer using PBE0 in the Gaussian code. Very similar values (4.153, 3.846, and 3.902 eV, respectively) of the HOMO–LUMO gap have been obtained using VASP with PBE0. This reflects the good accuracy of our calculations. There are large dipole moments on these capped rings, namely, 6.76, 8.03, and 9.08 D, respectively, for M = Ca, Sr, and Ba due to the charge transfer from the M atom to the C_9_ ring. These doped clusters have 38 valence electrons. Among these, two valence electrons in each carbon atom are covalently bonded with neighboring carbon atoms (with some deviation from a chain). This takes away 18 valence electrons from the C atoms. The remaining 20 delocalized valence electrons participate in bonding between the carbons and the M atom. As this delocalized charge lies mostly in the ring and between the ring and the M atom, we can consider a jellium model for a circular disk^26^ according to which clusters with 20 valence electrons correspond to an electronic shell closing and are magic. Therefore, the large HOMO–LUMO gap in these capped clusters is due to an electronic shell closing leading to the high stability of these species as it was also found^25^ in the case of a Be doped C_9_ ring cluster. Furthermore, it was shown that this ring cluster has π aromaticity with 10 valence electrons corresponding to the 4*n*+2 (*n* = 2) Hückel rule. We analyzed the energy levels and the molecular orbitals (MOs) in the case of CaC_9_, and its energy spectrum with MOs is shown in Figure S1. It is found that the spectrum has similarity with that of the Be@C_9_ case,^25^ and it also satisfies Hückel’s π aromaticity rule of 4*n*+2 (*n* = 2) corresponding to 10 valence electrons.

On the other hand, substitution of a divalent atom in place of a C atom in a C_10_ ring leads to a necklace shaped structure (Figure 2d–f) with 2 μ_B_ magnetic moments, which lie 1.199 (0.743), 0.907 (0.020), and 0.843 (0.173) eV higher in energy, respectively, for Ca, Sr, and Ba than the capped ring isomer using PBE (PBE0) in VASP. All the atoms in the ring lie nearly in a plane with small deviations. The chain isomer lies 2.072 (Ca), 1.820 (Sr), and 1.636 eV (Ba) higher in energy using PBE than the capped ring isomer. In the C_10_ ring isomer, 20 valence electrons are used in σ bonding between the neighboring C atoms while the remaining 20 delocalized valence electrons again form an electronic closed shell and make it magic. Accordingly, MC_9_ with M as a divalent atom has 38 valence electrons, which are two electrons short of electronic shell closing leading to 2 μ_B_ magnetic moments. Therefore, clusters with 38 valence electrons may favor a capped ring structure, while those with 40 valence electrons may favor a ring structure with M incorporation. Deviation from this number of valence electrons could give rise to magnetic moments on these clusters. We also tried capping an Mn atom on a C_9_ ring, and the optimized structure shows it to have 5 μ_B_ magnetic moments due to the five 3*d* unpaired electrons while the remaining two valence electrons occupy the LUMO of the C_9_ ring. The optimized structure is similar to that shown in Figure 2a. However, the other ring isomer with the Mn atom in the ring lies 1.015 eV lower in energy, and it has 3 μ_B_ magnetic moments as four valence electrons on the Mn atom are used in bonding in the ring. The atomic structure is like that shown in Figure 2d. The magnetic moments are mostly localized on the Mn site, which has 4.27 μ_B_, while four C atoms have about 0.26 μ_B_ magnetic moment each and five C atoms have −0.42 to −0.53 μ_B_ magnetic moment using PBE. There is a reflection symmetry in a plane bisecting the necklace in two halves perpendicularly and passing through the Mn atom. The Mn–C bond length is 1.914 Å, while the C–C bond lengths are 1.273, 1.323, 1.277, and 1.304 Å. Within the ring, there is antiferromagnetic coupling with −0.43, 0.26, −0.46, 0.25, −0.54, 0.26, −0.46, 0.26, and −0.43 μ_B_ magnetic moments cyclically on the carbon atoms. The HOMO–LUMO gap is 1.0 eV (2.732 eV) by using PBE (PBE0).

**Figure 3:**
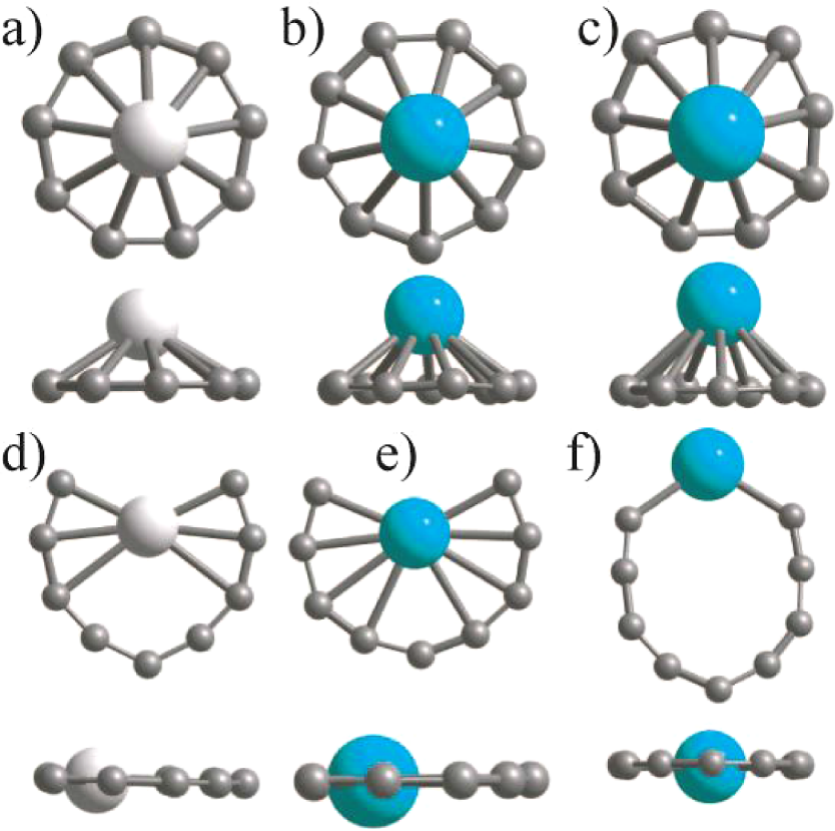
Capped ring and ring structures of cations of (a,d) ScC_9_, (b,e) YC_9_, and (c,f) LaC_9_, respectively. The capped ring structure has the lowest energy. Both the top and side views are shown. The large atom is the M atom.

**Figure 4:**
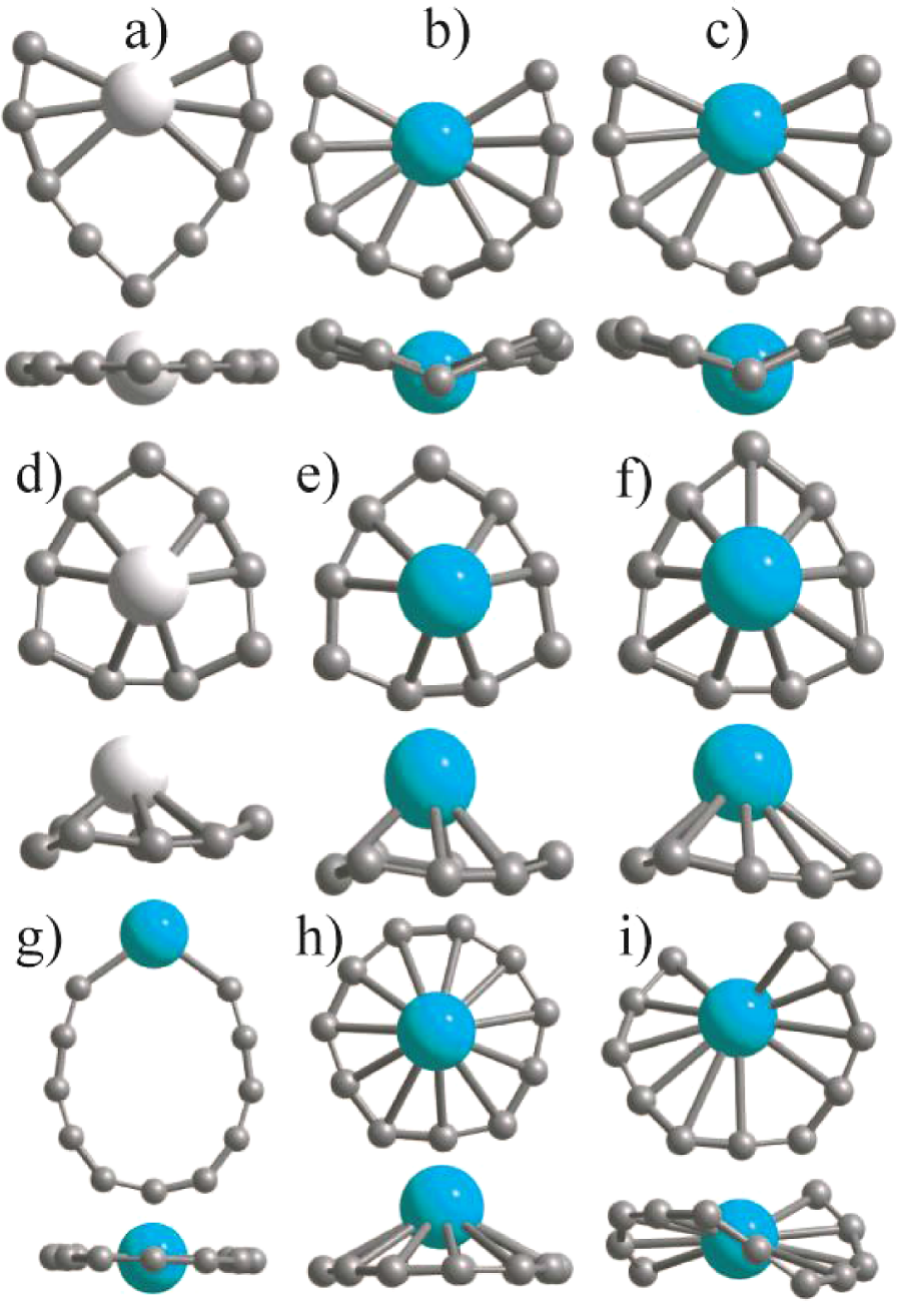
Ring and capped ring structures of anions of (a,d) ScC_9_, (b,e) YC_9_, (c,f) LaC_9_, (g,h) LaC_11_ cation, and (i) LaC_11_ anion. The ring structures have the lowest energy for the MC_9_ anions. For the LaC_11_ cation, the ring structure (Figure 4g) with the La atom in the ring has the lowest energy with PBE0 in Gaussian, but using PBE in VASP, the capped ring (h) lies slightly lower in energy. In Gaussian, the capped ring structure becomes distorted and lies slightly higher in energy. Both the top and side views are shown. One can see from the side view that the ring becomes slightly distorted in the capped ring isomer for the MC_9_ anion, while for the LaC_11_ anion (i), the ring is open.

Further calculations on cations of rings doped with Sc, Y, and La atoms show that these species being isoelectronic to Ca, Sr, and Ba doped C_9_, respectively, favor capping of the C_9_ ring by the M atom as shown in Figure 3. In the case of ScC_9_ cation, the ring becomes very nearly circularly symmetric (Figure 3a) with the mean C–C and C–Sc bond lengths of 1.307 and 2.227 Å, respectively, while for the YC_9_ (LaC_9_) cation, the nearest neighbor C–C and C–M bond lengths lie in the ranges of 1.287–1.325 Å (1.274–1.338 Å) and 2.377–2.408 Å (2.546–2.598 Å), respectively, using PBE0 in Gaussian program. The atomic structures remain similar when optimized using PBE and PBE0 except for a small contraction in the bond lengths when PBE0 is used. There are large HOMO–LUMO gaps of 4.601, 4.607, and 4.547 eV for ScC_9_^+^, YC_9_^+^, and LaC_9_^+^, respectively, using PBE0. The cation of Sc, Y, and La doped C_10_ like ring lies 2.738, 2.021, and 1.694 eV higher in energy than the capped ring isomer using PBE0 in the Gaussian program. The C_10_ like ring relaxes to a necklace structure and has 2 μ_B_ magnetic moments. Note that the MC_9_^+^ (M is a trivalent atom) ring has two valence electrons less than those in C_10_, which gives rise to 2 μ_B_ magnetic moments and a smaller HOMO–LUMO gap. On the other hand, anions of Sc, Y, and La doped species interestingly favor the C_10_ like ring (see Figure 4) and they lie 0.185, 0.716, and 0.885 eV lower in energy than the capped C_9_ ring isomers, respectively. These results show that the stability of the necklace isomer increases as one goes down in the group IIIA column in the periodic table. In both C_10_ neutral and MC_9_ (M = trivalent atom) anion cases, the number of valence electrons is the same. The M doped carbon ring relaxes to a necklace shape (Figure 4a) or a butterfly shape (Figure 4b,c), and the M atom drifts inside the ring (Figure 4a–c). There are large HOMO–LUMO gaps of 2.957, 3.114, and 2.726 eV, respectively, for M = Sc, Y, and La doped anions using PBE0, and in all cases, the magnetic moment is zero. The HOMO–LUMO gap is smaller than the values for the capped ring cations. The substitutional doping of an M atom in C_10_ reduces its symmetry as well as the HOMO–LUMO gap (5.70 eV) and brings the latter to the visible range. Accordingly, these M doped species become optically interesting. The C–C (C–M) bond lengths vary in the range of 1.271–1.356 (2.206–2.328 Å), 1.258–1.381 (2.404–2.579 Å), and 1.259–1.376 Å (2.556–2.736 Å) for MC_9_ anions, where M = Sc, Y, and La, respectively. The carbon ring in the capped ring isomer is slightly distorted, as shown in Figure 4. All the lowest energy isomers have no imaginary frequency and are dynamically stable. This has been further checked for the CaC_9_ case using ab initio molecular dynamics calculations by heating to 400 K, and the capped ring structure does not change much except for some displacement of atoms. It is to be further noted that the necklace shaped ring isomer has two structures: 1) with the M atom lying in the ring as shown in Figure 3f for LaC_9_^+^ where the M atom interacts with two C atoms and 2) where the M atom drifts inside the ring and interacts with more than two C atoms as shown in Figure 3d,e for M = Sc and Y, respectively, as well as in Figure 4a–c.

**Figure 5:**
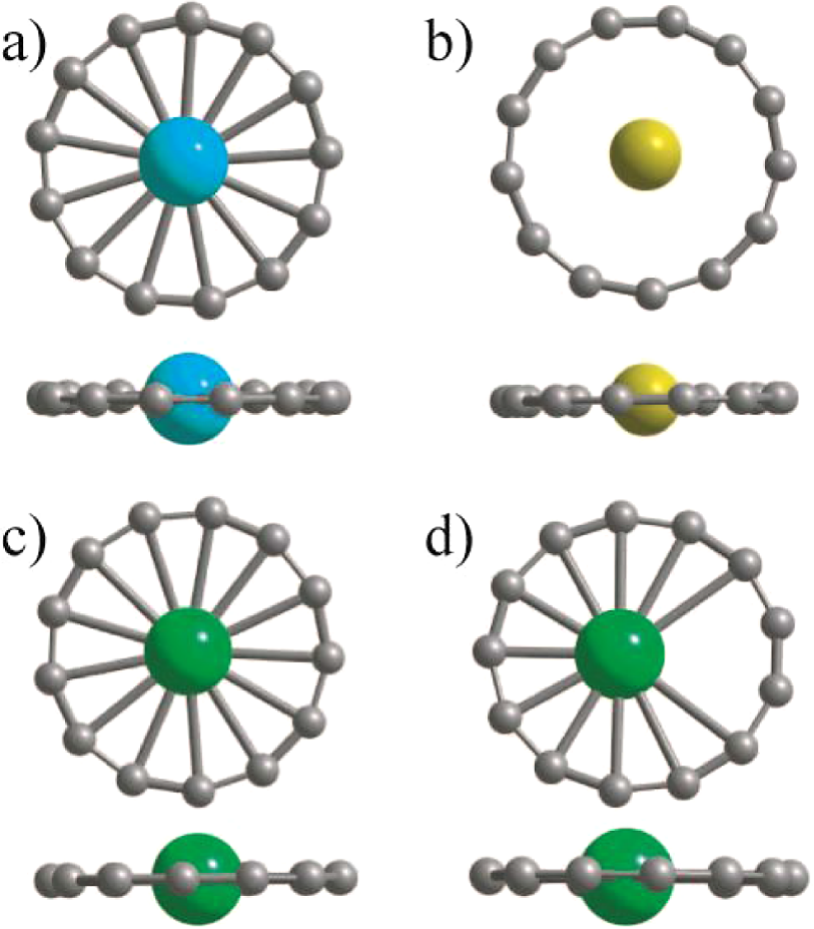
Optimized structure of (a) the La@C_13_ cation using PBE0 in VASP. It remains very similar when PBE is used. The side view shows that La lies in the plane of the ring. Similar results were obtained by using Gaussian and PBE0. (b) The atomic structure for neutral Ca@C_13_ and Sr@C_13_ as well as for Sc and Y doped cations in Gaussian with PBE0. The bonds between the M and C atoms have not been connected. (c) The cation of Gd@C_13_ with Gd at the center of the ring and in the plane of the ring. This is obtained when PBE is used in the VASP. (d) Optimized structure when PBE0 is used in VASP. The Gd ion is slightly displaced from the center though it lies in the plane of the ring.

As the size of the M atom is large, the ring of carbon atoms is partial in some cases, i.e., open, and the M atom interacts with many C atoms. These results suggest that we can add C atoms to complete the carbon ring. We added two C atoms in the case of LaC_9_ to form LaC_11_, and the optimization of the cation using PBE0 in Gaussian code shows (Figure 4g) that a structure with La atom in the ring has the lowest energy while a capped ring (Figure 4h) lies slightly lower in energy using PBE in VASP. Both have 2 μ_B_ magnetic moments. In the case of the LaC_11_ anion, the carbon ring is open and distorted as shown in Figure 4i. Therefore, La is still too large for the C_11_ ring to be accommodated inside it. Subsequently, we added two more C atoms to form an M@C_13_ ring in which the La atom is well accommodated endohedrally as shown in Figure 5a. This beautiful wheel-shaped cation cluster is a singlet as the two valence electrons from La^+^ occupy the two down spin states of C_13_ giving rise to a large HOMO–LUMO gap. The C–C bond lengths are nearly the same as in the undoped C_13_ ring, and therefore, there is little distortion from the doping of the M atom. This M doped ring has effectively 28 delocalized valence electrons while 26 valence electrons on C atoms are involved in σ bonding between the neighboring C atoms. Clusters with 28 delocalized electrons are magic in a disc jellium model^26^ and are more stable than the neighboring sizes. Accordingly, this ring should exist in a high abundance. We find that there is a large HOMO–LUMO gap of 2.770 eV (2.908 eV) within PBE0 using VASP (Gaussian), which suggests this ring to be good for visible light emission. The C–C bond lengths are all equal to 1.289 Å (1.291 Å), while C–La bond lengths have the value 2.694 Å (2.699 Å) in VASP (Gaussian). All of the vibrational frequencies are real, and this suggests that the ring structure is dynamically stable. Our results are in general agreement with the finding^24^ of ring isomers of La doped cation clusters. We further doped Ca, Sr, and Ba atoms to form neutral Ca@C_13_, Sr@C_13_, and Ba@C_13_ rings. The converged ring structures are also circularly symmetric (Figure 5b), but Ba lies above the ring due to its large atomic size. There is a large HOMO–LUMO gap of 4.448, 4.410, and 4.317 eV, respectively, suggesting their chemical stability.

**Figure 6:**
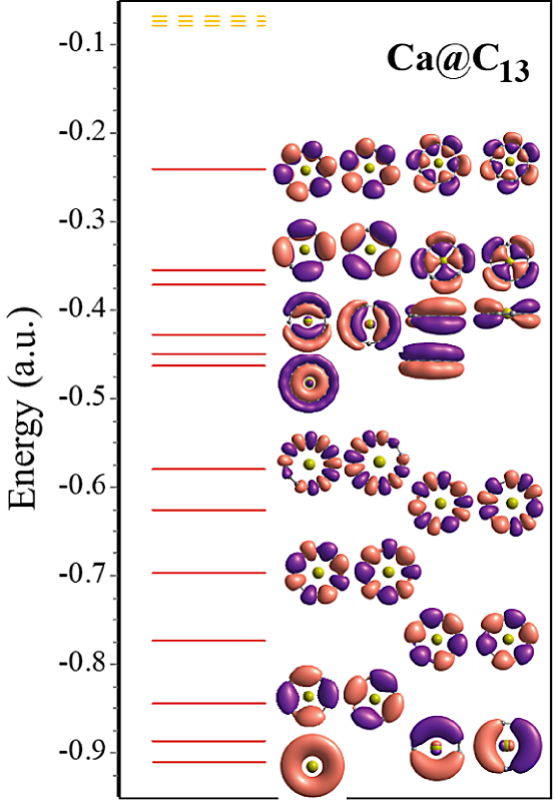
Valence electronic states (full lines) of the Ca@C_13_ ring. The corresponding occupied MOs are also shown. These can be labeled as 1S, 1P_*x*_, 1P_*y*_, 1D_*xy*_, , , , , , , , , , 2S, 1P_*z*_, 2P_*x*_, 2P_*y*_, 1D_*xz*_, 1D_*yz*_, 2D_*xy*_, , , 1F_*xyz*_, , , , and  type starting from the lowest state. Except for the 1S, 2S, and 1P_*z*_ electronic states, other occupied states are doubly degenerate. The unoccupied states are shown with broken lines.

In Figure 6, we have shown the electronic energy levels and the MOs of the Ca@C_13_ ring. Considering the *z* axis to be normal to the ring, the ordering of the occupied MOs is 1S, 1P_*x*_, 1P_*y*_, 1D_*xy*_, , , , , , , , , , 2S, 1P_*z*_, 2P_*x*_, 2P_*y*_, 1D_*xz*_, 1D_*yz*_, 2D_*xy*_, , , 1F_*xyz*_, , , , and . Accordingly, there are seven π-bonded MOs with 14 valence electrons. Therefore, this ring satisfies the 4*n*+2 (*n* = 3) Hückel’s rule of aromaticity. We have also shown the angular momentum and site projected density of states obtained from the VASP calculations using PBE0 for this ring in Figure S2. One can see that the low-lying states arise from the 2*s* atomic orbitals of carbon atoms while the higher lying states arise from the 2*p* atomic orbitals of carbon atoms. There is no significant contribution from the Ca atom in the occupied region, suggesting nearly complete charge transfer to the carbon ring.

**Figure 7:**
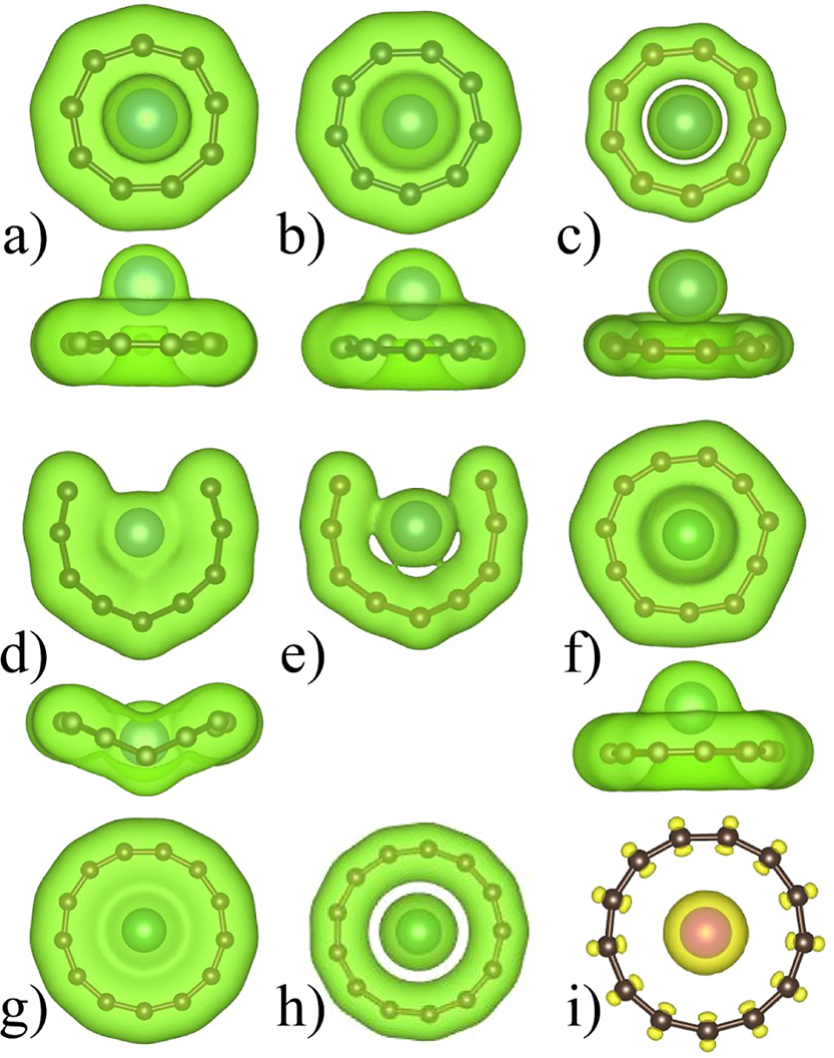
Charge density isosurface for (a) CaC_9_ (0.025 e/Å^3^), (b) YC_9_ cation (0.025 e/Å^3^), (c) YC_9_ cation (0.1 e/Å^3^), (d) YC_9_ anion (0.025 e/Å^3^), (e) YC_9_ anion (0.05 e/Å^3^), (f) LaC_11_ cation (0.025 e/Å^3^), (g) La@C_13_ cation (0.025 e/Å^3^), and (h) La@C_13_ cation (0.05 e/Å^3^). (i) The spin polarization isosurface (0.0025) in the Gd@C_13_ cation. An increase in the value of the charge density isosurface does not give any charge between the ring and the M atom indicating low density in this region. There is high density on the carbon ring and around the M atom. The latter arises due to the core electrons. In Figure 7a–d,f, both the top and side views are shown.

We further doped the C_13_ ring with a Gd atom and the Gd@C_13_ cation ring remains stable as shown in Figure 5c although the Gd ion gets very slightly displaced from the center of the ring using PBE. The displacement from the center is more significant when PBE0 is used (Figure 5d). This also creates some variation in the C–C bond lengths, which lie in the range of 1.282–1.294 Å. The C–Gd bond lengths are in the range 2.454–3.030 Å. There is a large magnetic moment of 7 μ_B_, which is mostly localized on the Gd ion (see Figure 7i) as the seven up spin 4*f* electrons on Gd remain unpaired except for some hybridization effects with the carbon ring. The up-spin and down-spin densities of states of this ring are also shown in Figure S2. Comparing this with the density of states for Ca@C_13_ (Figure S2), one finds that the spectrum of Gd@C_13_ is similar to that of Ca@C_13_ except for the seven 4*f* up-spin states of Gd while the down-spin 4*f* states lie above the HOMO. The doubly degenerate spin states at −4.14 eV have 2D character, and there is hybridization of the 5*d* atomic orbitals of Gd with the 2D MOs of the ring resulting in a shift to higher binding energies compared to the case of Ca@C_13_ but the hybridization with other occupied states is quite small. There is a large HOMO–LUMO gap of 3.353 eV within PBE0, and therefore, we call it a magnetic superatom ring. There is about 1.2 e of charge transfer from Gd to the carbon ring. Such magnetic superatoms have been predicted^27,28^ earlier for Mn@Sn_12_, Gd@Au_15_, and some other cases.^7^ They should also exist with different spins if other rare earth atoms are doped similar to the case of the doped silicon clusters.^29^

In Figure 7, we have shown the charge density isosurface for a few representative cases such as the CaC_9_ capped ring (Figure 7a), YC_9_^+^ capped ring (b), YC_9_^–^ C_10_-type ring (d), LaC_11_^+^ capped ring (f), and La@C_13_^+^ endohedral ring (g) using VASP with PBE. Also, in Figure 7c,e,g, we have shown the charge density isosurface for the YC_9_^+^ capped ring, YC_9_^–^ open ring, and LaC_13_ cation with a higher value (0.1, 0.05, and 0.05 e/Å^3^, respectively) of the isosurface. It is seen that there is a low charge density between the ring and the M atom. For the CaC_9_ case, there is little valence charge on the Ca ion due to the charge transfer to the C_9_ ring while high density of charge is there between the C ions. Note that we have considered 3*p* core electrons of Ca as valence electrons, and therefore, some charge is seen around the Ca ion. Similar results have been obtained for Sr and Ba capped rings as well as for the YC_9_ cation (Figure 7b,c). For the YC_9_ anion ring isomer also, there is covalent bonding between the neighboring C atoms and low charge density between the C atoms and M atom (Figure 7e). The remaining valence electrons are delocalized on the necklace and between the carbon necklace and the Y ion. Furthermore, in the case of La@C_13_^+^, the ring is nearly circular, and as shown in Figure 7g,h, there is covalent bonding between the nearest neighbor C atoms with high charge density so that one can consider 26 valence electrons to take part in it. The remaining 28 valence electrons are delocalized on the ring and between the ring and the M atom. In the case of Gd@C_13_^+^, the bonding character is similar to that in the case of La or Ca doped ring, but there are seven unpaired 4*f* electrons that give rise to spin polarization (Figure 7i), which can be seen to be mostly localized on the Gd ion. We further calculated the interaction of a ring with an M atom and the energy gained by the doping of an M atom, E_DE_, has been calculated from
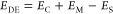
1where *E*_C_ and *E*_M_ are the energies of the carbon ring/chain and the M atom, respectively, while *E*_S_ is the energy of the M atom doped system. We find that there is a gain of 5.009, 4.682, 5.193, and 3.562 eV for the doping of Ca, Sr, Ba, and Mn atoms compared with the ground state of C_9_, the chain isomer. This energy gain increases further to 6.296, 6.292, and 7.206 eV for the doping of Sc, Y, and La atoms, respectively, within PBE. For the C_13_ ring, the doping energy becomes 5.991, 5.953, 6.348, 6.826, 7.176, and 8.083 eV for the doping of Ca, Sr, Ba, Sc, Y, and La atoms, respectively. The large gain in energy is responsible for the transformation of a C_9_ chain to a ring as well as further stabilization of the C_13_ ring. The adiabatic ionization potentials of M@C_13_ (M = Sc, Y, and La) are 6.474, 6.109, and 5.444 eV while the adiabatic electron affinities are 1.722, 1.631, and 1.873 eV, respectively. Large ionization potentials and low electron affinities are characteristic of magic clusters.

Bader charge analysis of the clusters was performed to further understand the bonding character in these doped rings. In the case of Ca, Sr, and Ba capped C_9_ rings, there is ∼1.5 e charge transfer from the M atom to the carbon ring. The charge is not equally distributed on all of the C atoms in the ring. Three C atoms have about 0.66 e excess charge, while one C atom has 0.34 e excess charge. The remaining five C atoms give about 0.09 to 0.32 e to neighboring C atoms. Therefore, there is a weak ionic bonding character within the ring and strong ionic bonding between the M atom and the ring. A similar behavior is seen for the cations of the Sc and Y capped rings. For ScC_9_^+^, there is 1.82 e charge deficiency on Sc while seven C atoms have small excess of charge (0.04 to 0.21 e), and two C atoms have a slight (0.02 and 0.1 e) deficiency of charge. Accordingly, the charge is dominantly removed from Sc for the cation, and the charge transfer to the ring is less compared with the doping of divalent atoms. Similar results are obtained for YC_9_^+^. In this case, there is a deficiency of 1.95 e on Y while a small charge (0.04–0.24 e) is transferred to seven C atoms and one C atom has a very slight depletion of charge (0.01 e). In these cases, about one valence electron remained on the metal ion. These effectively 20 delocalized valence electron species are also magic clusters.

**Figure 8:**
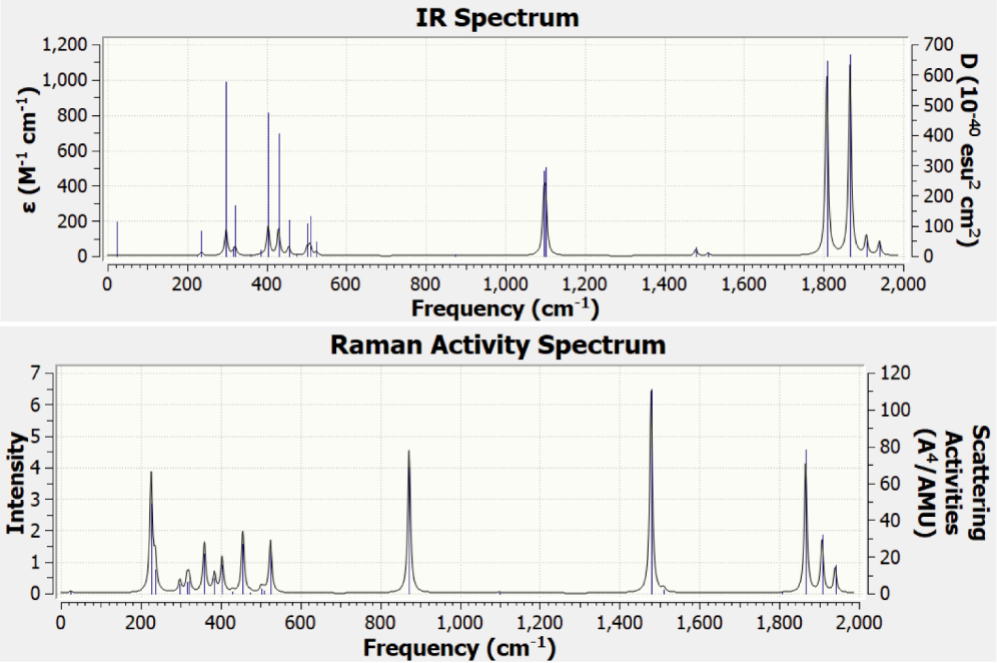
IR and Raman activity spectra for the Ca capped C_9_ ring cluster CaC_9_ with a Gaussian broadening of half width at a half-maximum of 4 cm^–1^.

In Figure 8, we have shown the Gaussian broadened IR and Raman spectra of CaC_9_ as a representative of the capped ring structure. The IR spectrum has two intense peaks at 1807.78 and 1866.08 cm^–1^, which correspond to the stretching of the C–C bonds. There are two weaker peaks at 1096.62 and 1101.12 cm^–1^, which correspond to bond stretching and the swing of the ring. The small peak at 297.99 cm^–1^ corresponds to breathing of the ring and up–down motion of the Ca atom. The Raman activity shows a peak at 225.81 cm^–1^, which is a bending mode, while a peak at 872.39 cm^–1^ corresponds to the breathing mode of the ring. The strongest peak at 1478.62 cm^–1^ arises from the C–C bond stretching. Another peak at 1866.08 cm^–1^ also corresponds to the C–C bond stretching. Similar results were obtained for SrC_9_ and BaC_9_. The IR spectrum for SrC_9_ has strong peaks at 1816.74 and 1839.65 cm^–1^ and two almost degenerate peaks at 1093.10 and 1093.43 cm^–1^. As these modes arise from the motion of carbon ions, the frequencies are similar in the case of CaC_9_. While the Raman activity shows a strong peak at 1507.88 cm^–1^ corresponding to C–C bond stretching. Another strong peak at 856.05 cm^–1^ corresponds to the breathing mode of the ring. The smaller peak at 229.76 cm^–1^ is the bending mode of the ring. For BaC_9_, the corresponding IR peaks are at 1820.46, 1852.73, 1092.57, 1093.38, and 222.13 cm^–1^ while the Raman activity peaks are at 1502.08, 854.23, and 224.64 cm^–1^. In the case of the YC_9_ cation, there are two prominent IR modes: 1) at 304.11 cm^–1^ and 2) a doubly degenerate mode at 1101.75 cm^–1^, which corresponds to C–C bond stretching. The Raman activity has a strong peak at 875.98 cm^–1^ corresponding to the breathing mode and two weaker peaks at 1522.69 and 1525.92 cm^–1^ corresponding to C–C bond stretching. For the LaC_11_ cation ring isomer (Figure 4g), the IR spectrum has strong peaks at 1938.78 and 1995 cm^–1^ both corresponding to C–C bond stretching and weaker peaks at 1666.88 and 1132.91 cm^–1^ corresponding to C–C bond stretching, and a peak at 321.89 cm^–1^ corresponding to the swinging motion of the ring. There is one very strong Raman active mode at 1938.78 cm^–1^ corresponding to C–C bond stretching. Other modes are much weaker. These results will be helpful in identifying the correct isomer in experiments.

**Figure 9:**
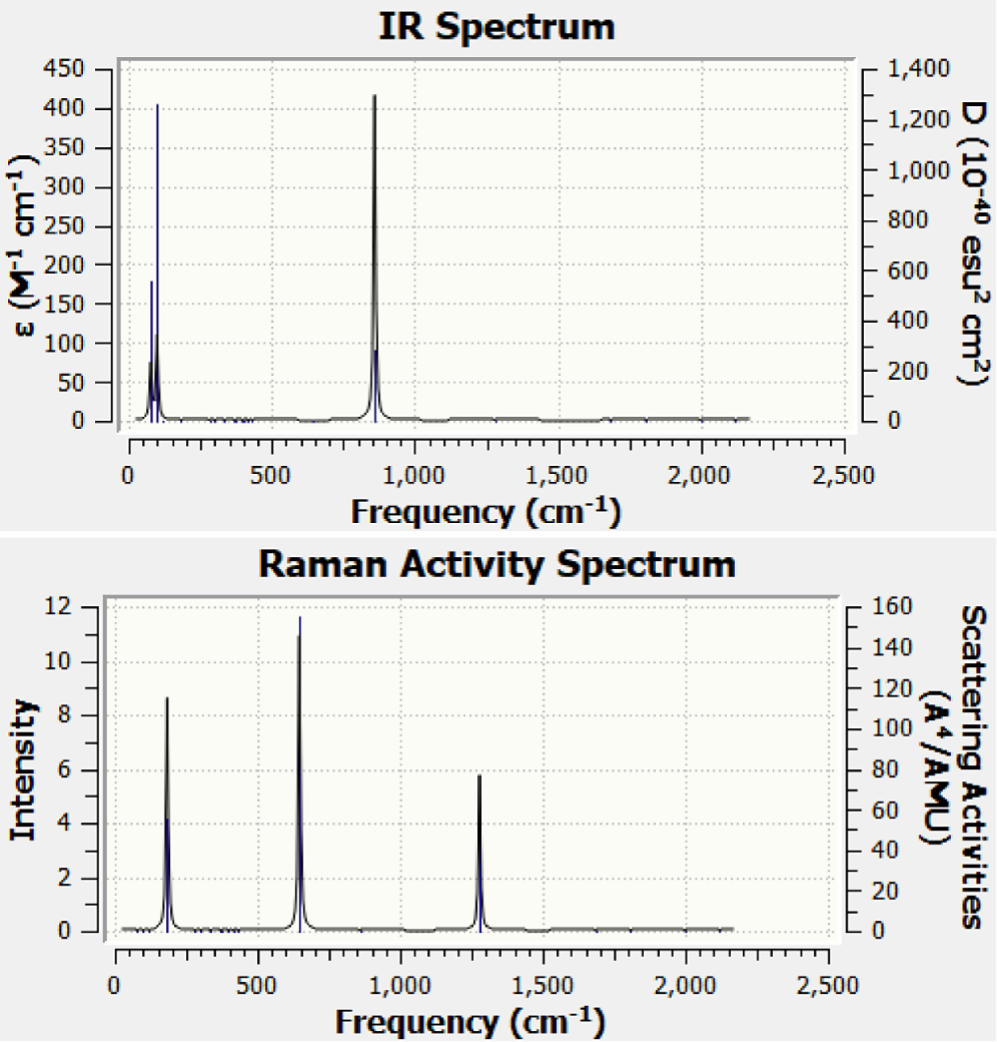
IR and Raman activity spectra for the Ca doped C_13_ ring cluster Ca@C_13_ with a Gaussian broadening of half width at a half-maximum of 4 cm^–1^.

The IR spectrum and Raman activity of the Ca@C_13_ ring are shown in Figure 9 as a representative of this ring structure. The high symmetry of the ring leads to only a few intense lines in the spectra. The IR spectrum has a degenerate strong mode at 860.25 cm^–1^, which is the stretching and swinging mode of the ring and does not involve motion of the M atom. There is a mode at 99.18 cm^–1^, which is the breathing mode of the M doped ring, while a doubly degenerate mode at 78 cm^–1^ is the swinging mode of the M atom and the ring. The highest Raman intensity at 647.24 cm^–1^ corresponds to the breathing mode of the ring. The doubly degenerate mode at 118.74 cm^–1^ is the bending mode of the ring while the doubly degenerate mode at 1278.23 cm^–1^ is the stretching mode of the ring. In all these modes, the M atom remains at its position.

## 3 Summary

In summary, we have reported the stabilization of ring structures of C_9_ with the doping of a metal atom. Divalent atoms such as Ca, Sr, and Ba as well as isoelectronic cations of Sc, Y, and La favor a capped C_9_ ring structure, while the anions of these trivalent atom doped clusters stabilize in a ring structure of MC_9_^–^ where the metal atom is part of the ring. These species correspond to an electronic shell closing with 20 delocalized valence electrons in a disk jellium model and have large HOMO–LUMO gaps. Also, following the Hückel 4*n*+2 aromaticity rule, there is π aromaticity with 10 valence electrons (*n* = 2). Further studies of the doping of a La atom have led to the findings of a necklace shaped ring of the LaC_11_ cation as well as a La@C_13_ ring cation with endohedral doping of a La atom in a wheel shaped C_13_ ring. The La@C_13_ cation ring has 28 delocalized valence electrons, which also correspond to an electronic shell closing in a disc jellium model. This ring also has π aromaticity with *n* = 3. Our results agree with the experimental report of La doped ring structures of carbon cation clusters in this size range and show for the first time their most favorable structures. Similar results have been obtained for Sc and Y doped cation clusters as well as for the divalent atom (Ca, Sr, and Ba) doped neutral clusters. We hope that these novel structures will stimulate further research on metal atom doped carbon clusters and other nanostructures. These species would be interesting for the study of single atom catalysis – a subject that is drawing great interest in recent years – as well as for understanding extended systems such as interaction of metal atoms with a defective graphene. Also, we found a magnetic superatom Gd@C_13_^+^ ring with 7 μ_B_ large magnetic moments (all up spin 4*f* states of Gd occupied) and a large HOMO–LUMO gap in which the Gd atom is endohedrally doped in a C_13_ ring. This could give a new direction to make the smallest magnetic species. Earlier magnetic slaved atoms have been shown in hydrogenated silicon cages^30^ and rare earth atom doped silicon cages with different magnetic moments.^29^ As the size of rare earth atoms are similar, we expect magnetic ring structures of carbon to have different magnetic moments with the doping of other rare earth atoms, and that our results will encourage researchers to find such species in experiments and further development of carbon-based smallest magnetic nanostructures.

## 4 Computational Method

The calculations have been performed using projector augmented wave pseudopotentials^31^ in Vienna Ab initio Simulation Package^32^ (VASP) and spin-polarized generalized gradient approximation^33^ of Perdew, Burke, and Ernzerhof (PBE) for the exchange-correlation functional. The clusters were placed in a large cube of side 20 Å, and the Brillouin zone integrations have been performed with only the Γ point. The structures were optimized without imposing any symmetry constraints and were considered converged when the absolute value of each component of the force on each ion became less than 0.005 eV/Å. The energy minimization was achieved with the tolerance of 10^–5^ eV. We also considered the effects of including Grimme’s D3 correction,^34^ but the contribution to the energy is about 0.08 and 0.126 eV for the lowest energy isomers of CaC_9_ and CaC_13_, respectively. The atomic structures are also not much affected. Therefore, we have not considered this in reporting results for different clusters. The obtained atomic structures and magnetic moments were further used as input for calculations with the PBE0 hybrid exchange-correlation functional in both VASP and Gaussian16.^35^ The latter was used to obtain IR and Raman spectra as well as to study charged clusters. We used the 6-311+G* basis set for the doping of Ca and Sc, and the QZVP basis set for the doping of Sr, Ba, Y, and La. The use of PBE0 results in a very small contraction in the bond lengths and improved the HOMO–LUMO gap compared with the results obtained with PBE. We give results obtained with both PBE and PBE0, but overall results with PBE0 are expected to be better than those obtained with PBE as the HOMO–LUMO gap is also underestimated using PBE. We also performed test calculations for the two ring isomers of CaC_9_ and CaC_13_ at the ωB97XD/def2-TZVP level^36,37^ of theory. The ωB97XD hybrid functional^36^ also includes Grimme’s D2 model.^38^ We found that the resulting capped ring structure of CaC_9_ is 0.489 eV lower in energy than the other ring in which Ca is part of the ring using ωB97XD/def2-TZVP as compared to 0.743 eV obtained by using PBE0/6-311+G*. Similarly, the endohedrally doped CaC_13_ ring lies 2.558 and 1.970 eV lower in energy than the other ring isomer with Ca in the ring using the PBE0/6-311+G* and ωB97XD/def2-TZVP levels of theory, respectively. Therefore, the energy difference between the two isomers is similar and the energy ordering is also the same. Further simulations have been done using ab initio molecular dynamics in VASP on CaC_9_ and Ca@C_13_ lowest energy ring isomers by heating at 400 K. After simulating for about 10 ps, we find that the clusters retain their structures. Therefore, the lowest energy structures are dynamically stable.

For the doped systems, a divalent M atom (Ca, Sr, and Ba) was doped in the chain and ring structures of undoped C_9_. For the chain isomer, the M atom was attached to one end of the chain while for the ring, we studied two isomers: 1) in which M atom caps the C_9_ ring as the M atoms studied here are too large to accommodate inside the ring, and 2) the M atom is a part of the ring as a C_10_ ring with one C atom replaced by the M atom. In the latter case, the carbon ring opens, and the M atom can either remain in the ring or drift toward inside the ring (endohedral) leaving the ring open. Therefore, further calculations were performed by adding C atoms to complete this ring. First, two C atoms were added to make LaC_11_. As the optimized ring structure of its anion was still open, two more C atoms were added to make the La@C_13_ cation. In this case, the M atom is endohedrally doped in a C_13_ ring. Therefore, further calculations were performed on isoelectronic cations of M@C_13_ (*M* = Sc, Y) and the Ca, Sr, and Ba doped neutral C_13_ ring. Also, Mn doped C_9_ and the cation of Gd@C_13_ have been studied to make magnetic species. Bader analysis has been performed to calculate the charge transfer between M and carbon atoms.

## Data Availability

The data that support the findings of this study are available within the article.

## Supporting Information Available

Electronic spectrum as well as MOs of CaC_9_ (Figure S1); Gaussian broadened total as well as angular momentum decomposed electronic DOS obtained from PBE0 calculation in VASP for Ca@C_13_ (Figure S2); the spin-polarized DOS for Gd@C_13_^+^ (PDF)
