# Supplementary material for: Ring Structures of Metal Atom Doped C9, C11, and C13 Clusters from Ab Initio Calculations—The Finding of a Gd@C13 Magnetic Superatom Ring
Source: ACS Omega. 2024 Jul 26;9(31):33919–27. doi: 10.1021/acsomega.4c04141 (PMC11308475; doi:10.1021/acsomega.4c04141)
Supplement: Supplementary file 1 — ao4c04141_si_001.pdf [file ao4c04141_si_001.pdf]

Supporting Information

Ring Structures of Metal Atom Doped C_9, C_{11}, and C_{13} Clusters from Ab Initio Calculations – The finding of a Gd@C_{13} magnetic superatom ring

Vijay Kumar*
Center for Informatics, School of Natural Sciences, Shiv Nadar Institution of Eminence Deemed to be University, NH-91, Tehsil Dadri, Gautam Buddha Nagar 201314, Uttar Pradesh, India
and Dr. Vijay Kumar Foundation, 1969, Sector 4, Gurgaon 122001, Haryana, India

*Corresponding author e-mail: Vijay.kumar@snu.edu.in

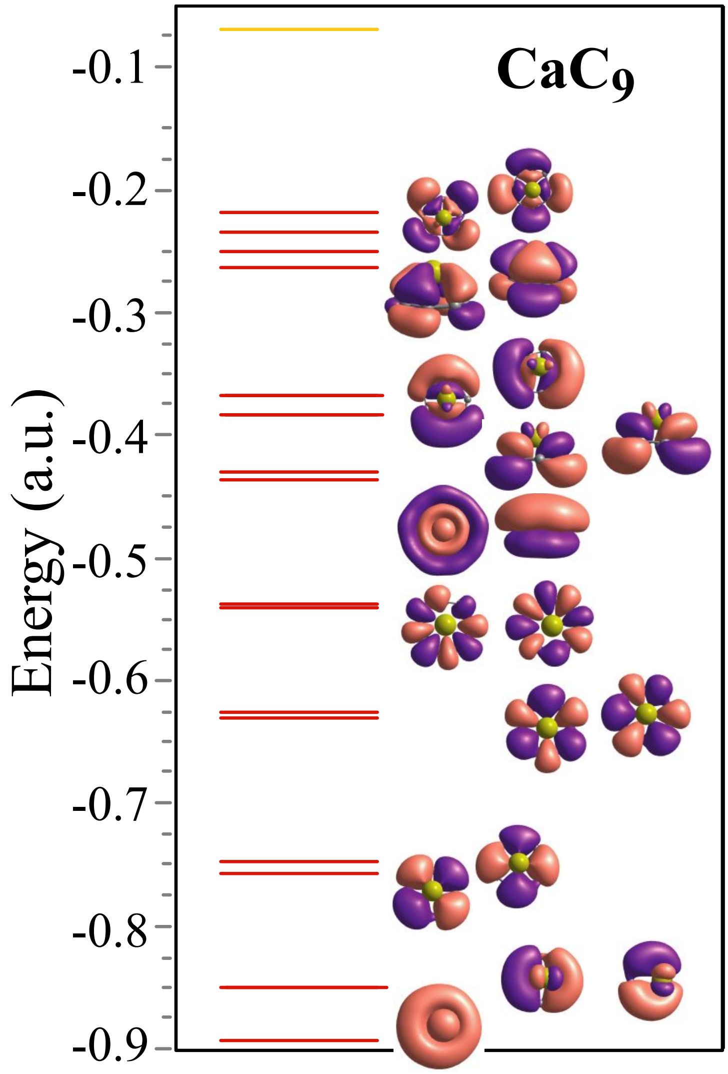

Figure S1. Electronic spectrum of CaC_9 isomer with Ca caping the C_9 ring using Gaussian 16 code with PBE0. Full (broken) lines are occupied (unoccupied) states. The corresponding molecular orbitals (MOs) are also shown. Considering the z axis to be normal to the ring, the ordering of the orbitals from the bottom to the highest occupied MO (HOMO) is 1S, $1P_x$, $1P_y$, $1D_{xy}$, $1D_{x2-y2}$, $1F_{x(x2-3y2)}$, $1F_{y(3x2-y2)}$, $1G_{xy(x2-y2)}$, $1G_{x4+y4}$, 2S, $1P_z$, $1D_{zx}$, $1Z_{yz}$, $2P_x$, $2P_y$, $1F_{z(x2-y2)}$,

$1F_{xyz}$, $2D_{xy}$, $2D_{x2-y2}$. There is a small distortion in the C_9 ring which lifts the degeneracy of the MOs. There are five π bonded MOs and therefore there is Hückel's aromaticity with 4n+2 (n = 2), 10 π bonded valence electrons.

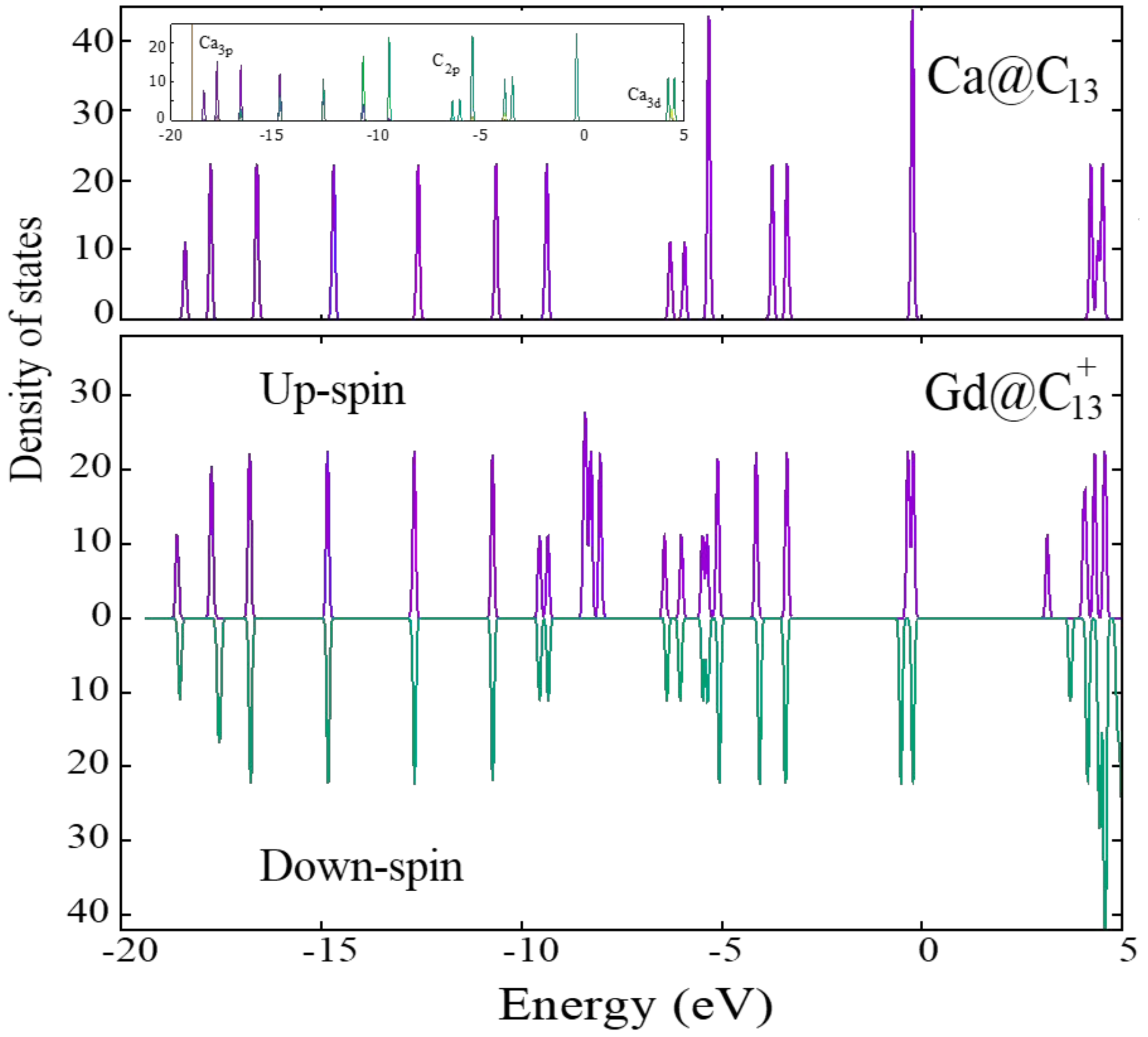

Figure S2. The electronic density of states (DOS) as obtained from VASP calculations using PBE0. The eigenvalues have been Gaussian broadened with a width of 0.05 eV. The upper panel is for Ca@C_{13} with no spin-polarization while the lower panel shows the up-spin (violet) and down-spin (green) DOS for Gd@C_{13}^{+}. The inset in the upper panel shows the site and angular momentum decomposed DOS for Ca@C_{13}. The sharp yellow line corresponds to the Ca 3*p* core states, the violet color shows the 2*s* states of C and green color shows the 2*p* states of C. There is nearly zero contribution from Ca valence electrons in the occupied region due to charge transfer. For the doping of Gd the two degenerate spin states at -4.14 eV have *d* angular momentum character and there is hybridization between the 5*d* states of Gd atom and the D type MOs of the carbon ring which also leads to a shift of these states compared with those for Ca@C_{13}. The hybridization with other states is rather very weak. The zero of energy is the HOMO.
